# Mining and visualizing large-scale course reviews of LMOOCs learners through structural topic model

**DOI:** 10.1371/journal.pone.0284463

**Published:** 2023-05-03

**Authors:** Linwei Yang

**Affiliations:** School of Foreign Languages, Yantai University, Yantai, Shandong, China; Tallinn University: Tallinna Ulikool, ESTONIA

## Abstract

Understanding Language Massive Online Open Courses (LMOOCs) learners’ subjective evaluation is essential for language teachers to improve their instructional design, examine the teaching and learning effects, and promote course quality. The present research uses word frequency and co-occurrence analysis, comparative keyword analysis, and structural topic modeling to analyze 69,232 reviews from one Massive Online Open Courses (MOOCs) platform in China. Learners hold a strongly positive overall perception of LMOOCs. Four negative topics appear more commonly in negative reviews as compared to positive ones. Additionally, variations in negative reviews across course types are examined, indicating that learners’ main concerns about high-level LMOOCs include teaching/learning problems, learner expectation, and learner attitude, whereas learners of low-level courses are more critical in the topic of scholarship ability. Our study contributes to the LMOOCs study by providing a better understanding of learners’ perceptions using rigorous statistical techniques.

## Introduction

The use of Massive Open Online Courses (MOOCs) has revolutionized the way people learn new languages. Language MOOCs (LMOOCs) are dedicated web-based online courses for foreign or second languages with unrestricted access and potentially unlimited participation [1], which provide an effective, convenient, and cost-effective way to acquire language skills, making them attractive to learners from diverse backgrounds. In recent years, the construction of LMOOCs has been booming dramatically on different MOOCs platforms worldwide. For instance, *iCourse*, one of the major MOOCs platforms in China, has launched more than 300 language courses. In *edX* and *coursera* platforms, there are more than 500 and 150 language courses respectively. Compared with traditional forms of language education, LMOOCs can create unlimited learning opportunities for language learners and provide innovative language learning experiences.

LMOOCs have been acknowledged to be an emergent and expanding research field [2–4], despite still being in their early developmental stage. Unlike traditional face-to-face teaching, LMOOCs platform is hard to instantly carry out communicative tasks, offer instructors’ feedback, and track learners’ explicit behaviors and potential psychological states, which has brought great challenges to further realize efficient language learning. A considerable literature has grown up and researchers have shown an increasing interest in the following topics and themes, including opportunities and challenges, case studies, pedagogical approaches, and the learners [5]. One of the key common underlying issues in current studies mainly lie in learners’ motivation, participation, and experience. Various investigations have discussed and tackled a wide variety of issues from learners’ perspectives. The first notable initiative in the study of LMOOCs learners is the exploration of participants’ motivation [6]. Later publications include self-directed learning or autonomy [7], the role of self-efficacy [8,9], engagement and participation [10], interaction [11], beliefs and attitudes [9,12], their perceived effectiveness [13], and multiple affordances [14]. These studies have provided valuable insights into different aspects of learners in LMOOCs, which can be used by instructors to better design and deliver high-quality courses, and help create effective online learning process and learner experience. However, most of them are case studies and draw their conclusions from traditional surveys in the form of questionnaires or face-to-face interviews. The survey may not be representative of the general population and the self-reported data may be subject to recall and social desirability bias. Learners’ overall perception of, belief in, attitude to, and satisfaction with LMOOCs (elements like the course quality, teacher quality, technical challenge, course content, etc.) is still less examined and the whole experiential profile is still unknown for LMOOCs researchers.

As learners have unique personalities, histories, motivations, interests, and identities [15], LMOOCs are supposed to provide tailored learning opportunities to their individual needs and preferences. Without considering learners’ interest, personalized demand, and attitude, LMOOCs will lead to cognitive load, learning boredom, and anxiety, and will end up with a negative learning experience. The increasing amount of learner-generated forum discussions and course reviews in MOOCs serve as a valuable data source for analyzing learners’ experiences, which entails their perception of the course, personalized demands, and their interaction with the instructor and other learners. Such data can be used to inform decisions about course design, instructor selection, and future curricular offerings and has become mainstream since the year 2018. For instance, general MOOCs researchers have frequently conducted sentiment analysis and topic modeling on comments from discussion forums [16], course reviews [17,18], and public responses to MOOCs on social media [19]. However, LMOOCs researchers prefer to use qualitative content analysis to explore learners’ subjective evaluation. Ye and Luo [20] explored how learners perceived LMOOCs by analyzing 600 comments and identified the key factors in their evaluation. Hsu [21] adopted a grounded theory method to examine learners’ course reviews from one course and proposed a conceptual model for the applicability of LMOOCs. Luo and Ye [22] analyzed 1000 evaluations using a qualitative method to examine the quality issues of LMOOCs and identified a framework in five aspects: teacher/instructor, teaching content, pedagogy, technology, and teaching management. While manual content analysis can provide valuable insights, it can be a labor-intensive and time-consuming task. Furthermore, it requires well-designed frameworks for interpretation and is heavily reliant on researchers’ expertise, which inevitably has subjective biases leading to potential issues with the reliability of coding results. The limited sample size might cause results to be with low generalization ability. Although numerous studies have made significant contributions to understanding various aspects of LMOOCs learners, a comprehensive and holistic understanding of the learner experience is still elusive.

The review above indicates that it is urgent for LMOOCs to adopt computer-aided techniques like frequency analysis, topic modeling techniques like Latent Dirichlet Allocation (LDA), and STM to automatically uncover the present state of learner experience as well as its influencing factors. However, the large-scale learner-generated textual data have been scantly mined in LMOOCs. To address this issue, Peng and Jiang [23] started to explore students’ sentiments and opinions through sentiment analysis and identified five major themes that encapsulated students’ opinions and experiences including attitudes, comments, evaluations of instruction and instructors, learning outcomes, and suggestions. Sentiment analysis like Peng and Jiang’s failed to distinguish between the positive and negative themes or topics, leading to a narrow conclusion. The traditional way of applying sentiment analysis may lead to the potential danger of mixing the positive or negative sides since reviewers usually do not complain or praise from the beginning to the end of writing a comment. To gain a comprehensive understanding of LMOOCs learners’ perceptions and opinions, it is important to identify topics that are significantly more prevalent in negative reviews than positive ones. STM is an appropriate method for this task as it enables researchers to incorporate external covariates that may impact topic distributions. Currently, there is no evidence of studies utilizing topic modeling, particularly STM, to extract LMOOCs learners’ subjective evaluations.

By this idea, this study utilized STM to mine learners’ opinion from LMOOCs course reviews to identify the main negative and positive topics and to reveal the variations in learner complaints across courses with varied types. To our knowledge, this is the first time to use STM model applied in LMOOCs research, which will contribute to LMOOCs learner research literature by (1) uncovering topics that frequently appear in both positive and negative reviews, and (2) exploring differences in learner complaints among courses with varied course types. This study aims to answer the following three questions: (1) What is learners’ overall perception of current LMOOCs in China? (2) What are the main topics Chinese LMOOCs learners are concerned about? (3) How do the topics vary among the courses with varied course types? The findings are supposed to shed light on LMOOCs design, development, and implementation.

## Materials and methods

### Data collection

LMOOCs have a salient presence in China MOOCs education due to its largest number of English language learners in the world. Additionally, students in colleges and universities are required to take national English language tests to obtain certificates. In light of this, several major LMOOC platforms have emerged. The most popular LMOOCs platforms are *iCourse* (https://www.icourse163.org/) and *UMOOCs* (https://www.unipus.cn/), which offer a wide range of online courses typically including video lectures, interactive quizzes, assignments, and discussion forums. Learners can choose courses for free, or pay a fee for a certificate of completion with personalized learning experiences, and flexible learning options. These platforms have received support from China’s Ministry of Education and have been quite popular among English language learners in China.

The study gathered 75,653 course reviews from 256 English language courses on the *iCourse* platform, a MOOC platform also known as Chinese Universities’ MOOCs. The *iCourse* platform was launched in 2011 by the Ministry of Education and the Ministry of Finance in China, offering free quality courses from renowned higher education institutions in China. In 2020, the platform offered more than 9,500 courses to over 9 million learners. The course reviews were collected by a self-built Python crawler from course review webpages using course ids. Each record in the dataset contains four features: the review text, course name, rating grade, and post time. The reviews were submitted between October 15, 2018, and November 26, 2020. All the details are listed in Table 1.

**Table 1:** Learners review record examples.

Review text	Course	Rating
It breaks the traditional teaching mode and highlights students’ autonomy, interaction and individualized learning.	*Academic English for Communication*	5
I benefit from it a lot. I think the course is very useful. It is very helpful to my English pronunciation. I am very happy to use this platform.	*English Pronunciation*	5
I have learned a lot and it’s worth it. Advice: the workload of assignments should be reduced. Thank you.	*English for Marketing and Sales*	4
You can do better with more correct pronunciation, but all in all, very good.	*International Writing and Writing*	4
The lecture is clear and complete, but we have too many assignments.	*Academic English for Communication*	3
The lecturer is excellent and provides detailed examination contents, but the answers to blank filling are a little bit rigid.	*Business English*	2
I have to rate one star and I cannot tolerate it for being maliciously given a low score by other students.	*Translation in Practice*	1

### Data preprocessing

The raw data collected consisted of 75,653 reviews, which included various forms of noisy text, such as meaningless numbers, repeated punctuations, and repeated single Chinese characters or English letters. However, reviews that contained repeated number “6” like “666” were considered valid data since they are widely recognized internet buzzwords with strong positive sentiment. After manually removing the nonsensical reviews, we were left with 69,232 reviews for further analysis. The majority of these reviews were written in Chinese, with a small portion in Chinese Pinyin or English. Based on the data, 82.25%, 12.77%, 2.79%, 0.75%, and 1.45% of the reviews were rated 5, 4, 3, 2, and 1 point(s) respectively, as shown in Table 2.

**Table 2:** Statistics of the raw and included course reviews.

Item	Frequency	%
1-star	972	1.40
2-star	508	0.73
3-star	1902	2.75
4-star	8854	12.79
5-star	56996	82.33

To conduct a word frequency-based analysis, we segmented and tokenized both Chinese and English reviews. We then divided the reviews into two main groups based on learners’ ratings: negative reviews with 1- or 2-point ratings, and positive reviews with 4- and 5-point ratings. We also categorized the courses into 12 different types (as shown in Table 3), including literature, linguistics, culture, translation, writing, grammar, speaking, and others, to facilitate the comparison of differences in learners’ evaluations. To examine the moderating role of course types, we coded these 12 types into three major levels: 1 for basic, 2 for intermediate, and 3 for advanced courses.

**Table 3:** Statistics of the course categories.

Level	Item	Frequency	%
1	listening	6	2.34
1	speaking	26	10.16
1	reading	5	1.95
1	general writing	14	5.47
1	grammar	6	2.34
1	Integrated course	71	27.73
1	IELTS	1	0.39
2	literature	20	7.81
2	culture	39	15.23
2	translation	12	4.69
3	English for Special Purpose	48	18.75
3	linguistics	8	3.13

### Tools and analysis

The main tool used for tokenization is PKUSEG [24] which is one of state of the art Chinese segmenting tools. To perform text mining and visualization, we used Quanteda [25] and Structural Topic Model (STM) [26] packages in R. Compared to traditional corpus analysis tools, R packages such as Quanteda and STM have four significant advantages: rich visualization capabilities, powerful natural language processing techniques such as topic modeling and semantic networks, high compatibility with other natural language processing tools, and fast running speeds for large-scale data. Additionally, we utilized two publicly available Chinese and English stop-word lists in our study.

To mine and visualize the opinion from learner reviews multidimensionally, this study took advantage of multiple natural language processing methods and statistical analysis. In addition to word frequency statistics and word cooccurrence network to show the most frequently used words across different rating groups and to visualize learners’ major concerns, two major techniques were performed in this study: (1) comparative keyword analysis to analyze the distribution difference of keywords [27]; (2) structural topic modeling [26] to identify the main topics and their correlation with course categories.

We employed the G-test of goodness-of-fit, also referred to as the likelihood ratio test or G2 test, to conduct comparative keyword analysis. The test compared the observed word frequency of observations in positive and negative rating groups with the expected counts. This statistical test serves as an alternative to the Chi-Square Goodness of Fit test and is commonly applied when dealing with large research data. The G statistic can be calculated using the formula (1):

G=2*∑[O*ln(O/E)] (1)

O: The observed count in a cell of a contingency table

E: The expected count in a cell of a contingency table

**Fig 1:**
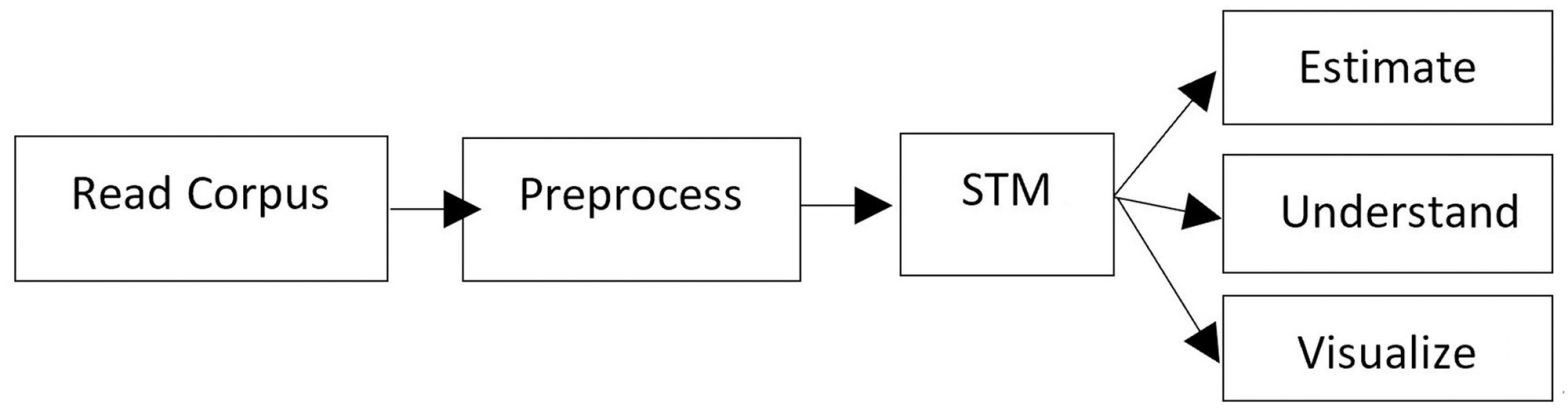
Heuristic description of stm package features in R.

The STM is an unsupervised learning model introduced by Roberts et al. [26] which has been widely used in the social science research field. One of the key innovations is that it allows researchers to integrate external metadata, defined as meta information about each document, into the topic model. By doing so, researchers can identify topics and estimate their relationship with the external variables, allowing for hypothesis testing about these relationships. Taking advantage of this feature, we utilized the STM to examine how positive and negative sentiments of course reviews, course types, and their interactions impact topic prevalence. To explore the influence of document-level covariates on topic prevalence, we defined negative and course labels as covariates, indicating review orientation and course ratings, respectively. Negative reviews were classified as those with 1- and 2-point ratings, while positive reviews had 5-point ratings. Course type labels ranged from 1 to 3, representing basic, intermediate, and advanced courses, respectively. The entire STM procedure (Fig 1) followed the methodology described by Roberts et al. [26].

## Results

### Most common words and co-occurrence network

Word frequency statistics (Table 4) showed that the most frequently used 50 words were mainly positive representing learners’ overall perception. It could be seen that learners preferred to comment on the elements of “teacher”, “course”, “content”, “learning”, “instruction” and “knowledge” and used words and phrases such as “good”, “clear”, “practical”, “interesting”, “easy to understand” and “vivid” to express their attitude and feeling. The mainstream opinion was that learners “gained” “a lot” in terms of “knowledge” and the courses were very “helpful”, thus they appreciated both the MOOCs and teachers.

**Table 4:** Statistics of the most common 40 words.

Word	TF	DF	Word	TF	DF
老师/teacher	15986	14385	视频/video	1364	1263
课程/course	12206	11095	感谢/thanks	1288	1190
学习/learning	8130	7250	生动/vivid	1284	1278
内容/content	7338	7074	东西/things	1261	1248
不错/good	5150	4852	写作/writing	1245	1078
知识/knowledge	3886	3732	教学/teaching	1177	1090
讲解/explain	3415	3304	提高/improve	1162	1122
收获/gain	3081	3069	有用/helpful	1162	1151
学到/get it	2785	2761	理解/understand	1125	1081
清晰/clear	2269	2239	时间/time	1091	996
实用/practical	1904	1885	细致/in detail	1079	1078
有趣/interesting	1877	1859	谢谢/thanks	1020	975
详细/details	1685	1681	易懂/easy to understand	1011	1011
希望/hope	1641	1592	推荐/recommend	992	970
讲课/teach	1625	1602	老师们/teachers	969	914
发音/pronunciation	1612	1492	充实/substantial	861	860
文化/culture	1599	1358	学生/students	856	760
授课/teach	1556	1494	基础/basics	835	773
受益匪浅/learned a lot	1466	1465	上课/attending class	801	770

**Fig 2:**
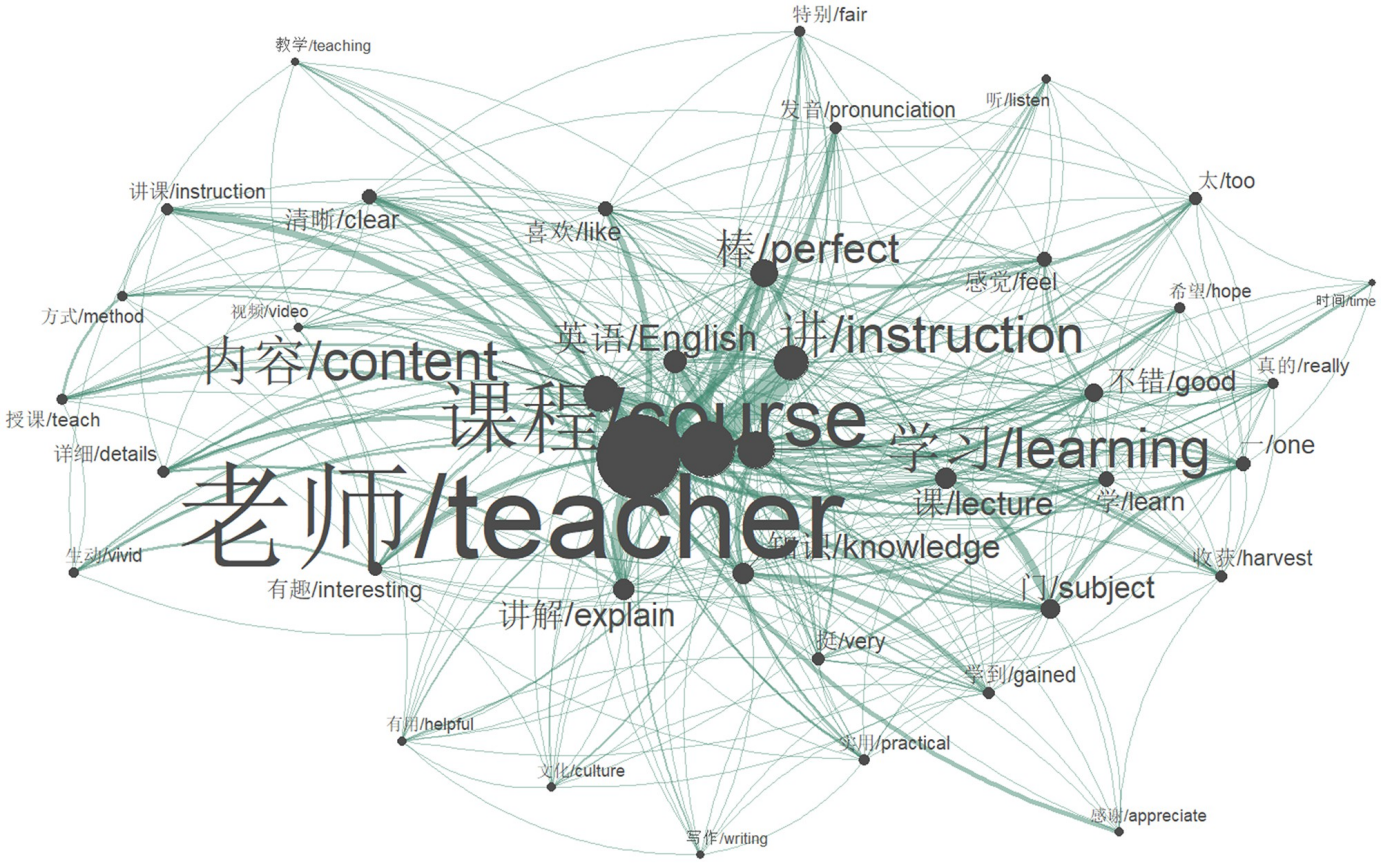
The co-occurrence network of the most common words.

To obtain a broader semantic context of the most frequently used words, a co-occurrence network was constructed, linking words based on their semantic relationships. The network’s structure is illustrated in Fig 2, where a node corresponds to a high-frequency word, an edge denotes a co-occurrence relationship between two high-frequency words, and the node degree indicates the node’s importance. The network reveals that learners’ reviews can be broadly categorized into three main themes: the teacher’s core elements, including instructional approach, voice, and pronunciation; the quality of teaching contents, learning materials, and videos; and learners’ attitudes and feelings towards the MOOCs. For instance, learners positively rated the teacher’s instruction and explanation as “clear” and “vivid”, and were pleased with the teacher’s pronunciation. The content and materials in MOOCs were regarded as “practical” and “systematic”, with writing and culture-related courses being more practical than others. Overall, learners reported a “good” experience and felt that they had benefited from the courses in terms of gaining knowledge and understanding the target language, culture, and translation.

**Fig 3:**
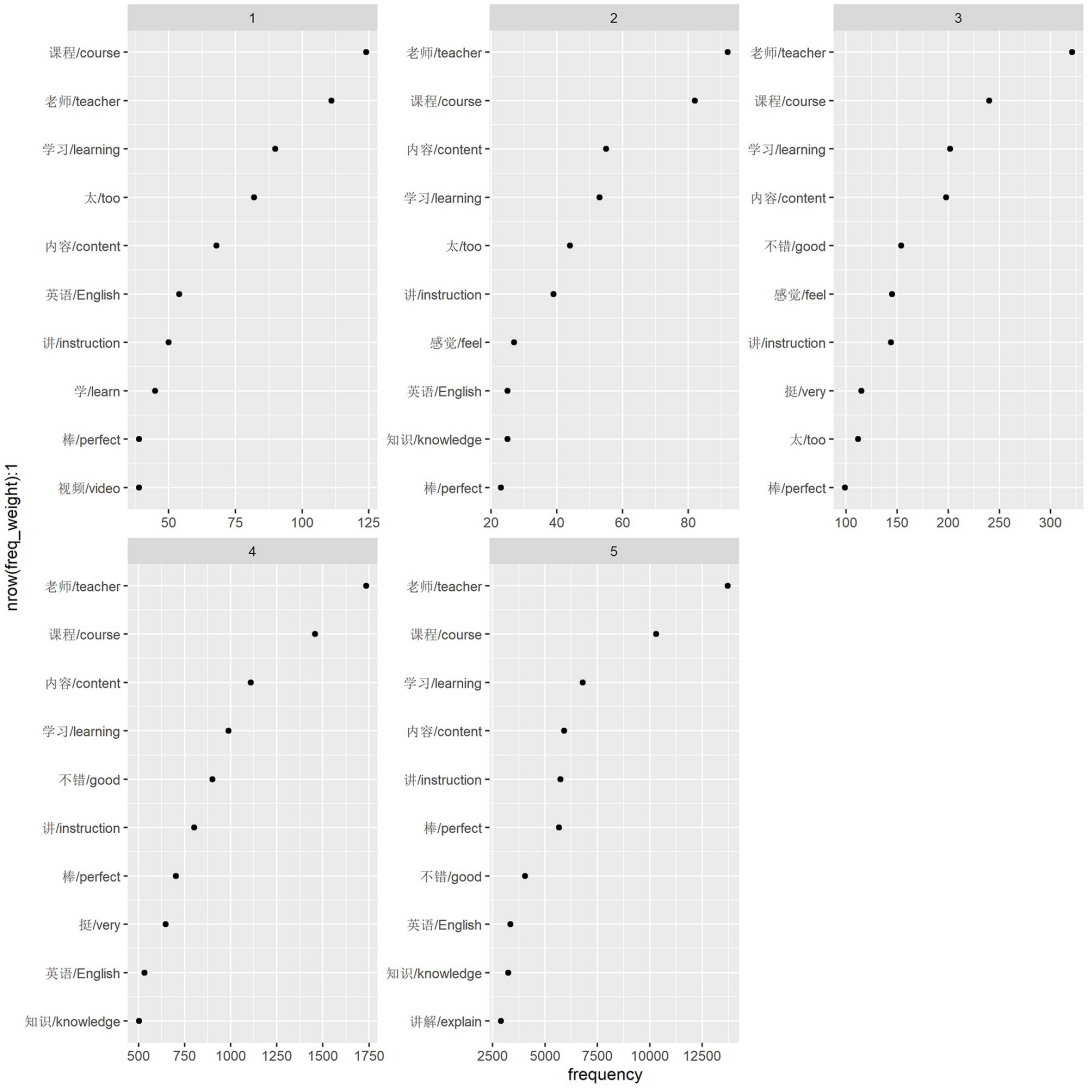
Top ten words for each rating group.

According to learners’ rating points (1–5 points), we divided the reviews into two groups, 5- and 4-point as positive and 2- and 1-point as negative. The top ten words’ distributions in Fig 3 showed that both positive and negative reviews share 70% of words in common, which were “老师/teacher”, “课程/course”, “内容/content”, “讲/instruction”, “学习/learning”, “英语/English” and “棒/perfect”. Four salient words in positive reviews were “知识/knowledge” and “讲解/explain” while in the negative group were “视频/video” and “太/too”. The salient negative words represented learners’ dissatisfaction with video-related techniques, which might be too difficult or of low quality. To verify the reliability of the visualization results, we considered those words and their closely related words as probes and conduct the G2 independence test. The results showed that the distribution of the above words differed significantly between positive and negative groups: “太/too” (G = 8.501, p = 0.000), “知识/knowledge” (G = 27.062, p = 0.004), “视频/video” (G = 320.268, p = 0.000), “难/difficult” (G = 459.349, p = 0.000), and “发音/pronunciation” (G = 919.083, p = 0.000).

**Fig 4:**
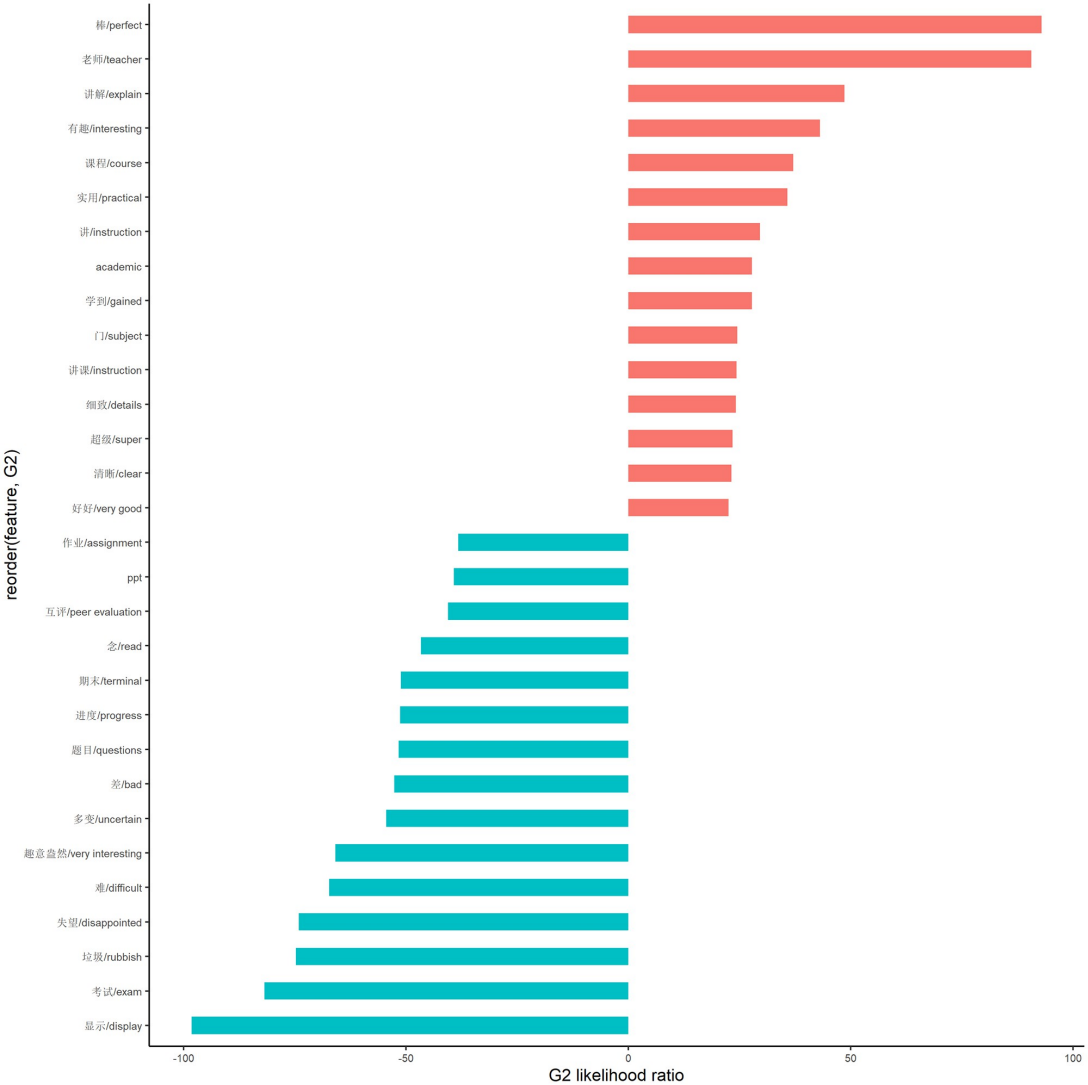
Comparative keywords analysis.

To further capture the full profile of learners’ positive and negative concerns, we conducted a keyword analysis to calculate the keyness of words by comparing their frequency and distribution in the positive and negative groups (Fig 4). The analysis revealed that learners who gave 4- and 5-point reviews spoke highly of their instructors and expressed great appreciation for them. In addition, they commented on learning a lot, especially in the writing and academic courses. Conversely, among the 1- and 2-point reviews, the negative evaluation is mainly related to the following aspects: (1) the display quality of courseware and video; (2) the final exam and test questions; (3) the time arrangement and teaching style of the course; (4) the overall perception of the MOOCs. For example, learners were disappointed and rate the MOOCs as “bad” and “rubbish”, because their teachers just read their lesson plan or “PPT”. They were also worried about peer evaluation and time arrangement, which affected their scores on quizzes and final exams. Notably, the positive Chinese idiom “very interesting/趣意盎然” was salient in negative reviews implying a strong ironical negation that the LMOOCs were too boring. Overall, the keyword analysis results provided teachers with a comprehensive understanding of learners’ learning experiences and needs.

### Topic identification for positive and negative reviews

According to scores on average semantic coherence and exclusivity, an optimized topic model (k = 12) with 12 topics was obtained from all the reviews. Table 5 shows the structural topic modeling results. Labels of the topics are listed in the first column, with the top terms having the highest frequencies and exclusivities of each topic. The third column indicates topic prevalence. The most concerning topics are learner experience and teacher quality, followed by learner improvement, scholarship ability, course quality, content quality, learner attitude, teaching method, learner expectation, teaching/learning problems, learner friendliness, and learner perception.

**Table 5:** Structural topic modeling result (k = 12).

Topic label	Representative terms (translated from Chinese)	%
learner friendliness	practical, understandable, systematic, clear, simple, easy, good	3.4
teacher quality	teacher, teaching, pronunciation, explanation, details, recommend, professional	14.6
teaching method	clear, instruction, vivid, interesting, teacher, well-organize, excellent	5.4
learner experience	learning, gain a lot, approach, experience, perfect, good, in general	19.9
learner attitude	like, good, great, recommend, wonderful, vision, benefit	6.2
course quality	course, too, super, arrangement, design, support, resource	8.1
learner improvement	culture, writing, improve, understand, English, translation, ability	13.4
content quality	content, very, interesting, substantial, meaningful, diverse, helpful	6.4
scholarship ability	difficult, writing, academic, lot, paper, teacher, future	8.7
teaching/learning problems	hope, time, assignment, video, subtitle, a little, test	5.2
leaner expectation	expect, think, speak, college, first time, course, MOOCs	5.4
learner perception	perfect, helpful, nice, 5-star, teachers, harvest, benefit	3.4

**Fig 5:**
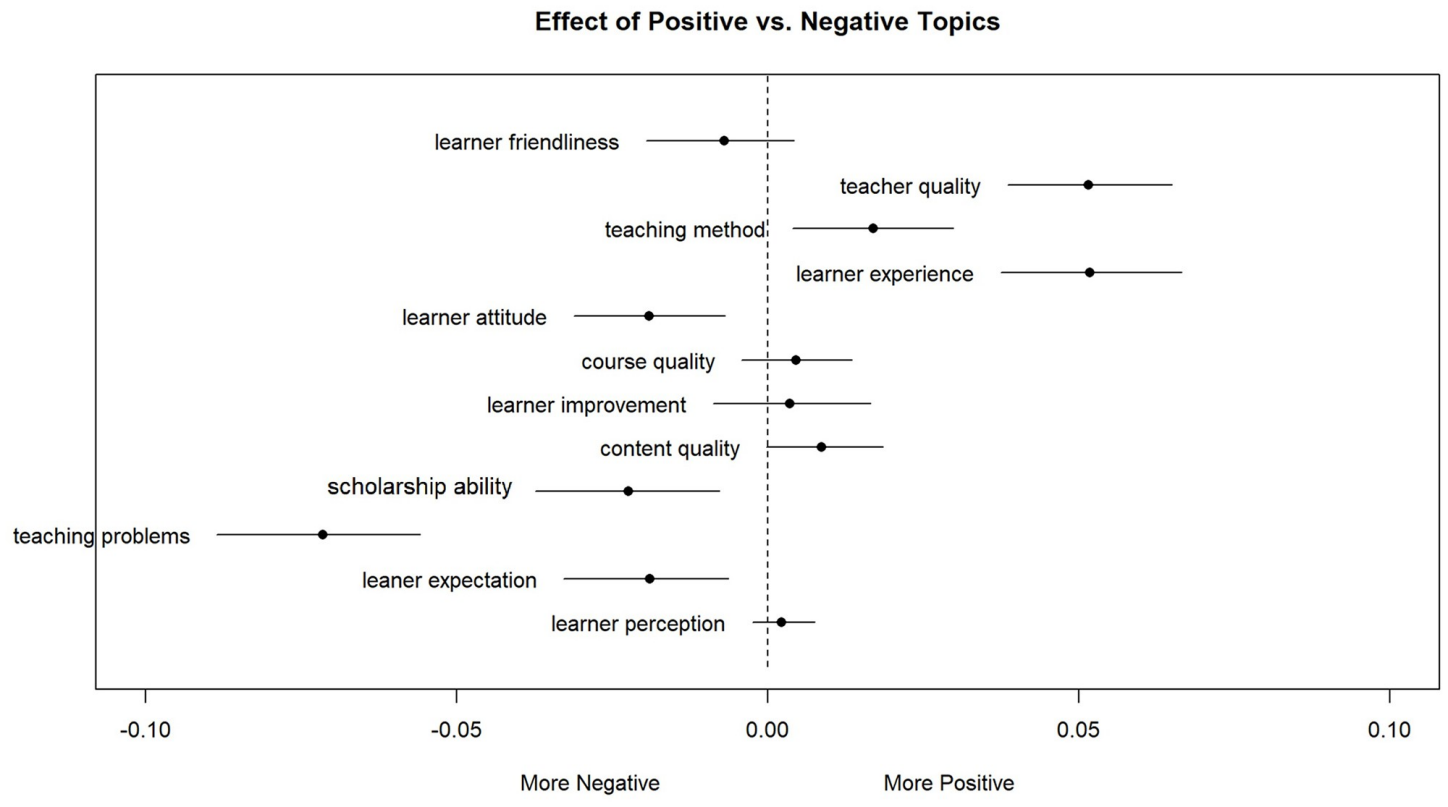
Differences in the negative topic prevalence.

Fig 5 visualizes the relative topic prevalence in both positive and negative reviews, where dots mean the mean values of the estimated differences and the bars are 95% confidence intervals for the difference. The results show four significant positive topics of teacher quality, learner experience, teaching method, and content quality. It also shows four major negative topics including teaching/learning problems, academic writing, learner expectation, and learner attitude.

The topic of teaching or learning problems ranks top one with representative words such as “hope”, “time”, “assignment”, “video”, “subtitle”, “test” and “a little”. The manual check of concordance lines with those keywords indicated that the topic was commonly associated with three aspects: the course arrangement concerning time and speed, the technical issues concerning video resolution and subtitles, and grading issues concerning assignments and exams being a burden or unfair. The second most negative topic is academic writing. Although a large portion of reviews was positive for its practicality and usefulness for learners’ future learning, the associated complaints center around academic papers’ difficulty as learning material and inappropriate difficulty level of exercises. Furthermore, some learners commented that instructors’ feedback on writing practices is too general and not helpful. The third topic is learner expectation with representative reviews from beginners. Some learners expected to learn more than what LMOOCs provided. Some were not willing to “express” their opinions or even do not know what to comment, which was a means of showing strong dissatisfaction. In addition, there were learners complaining that as they studied on the platform of LMOOCS for the first time they meet some technical problems such as broken links and lost downloaded material, which was out of their ideal expectations. The fourth topic, learner attitude, was slightly negative in Fig 5, but it was interesting that our manual examination of reviews lead to more of a positive topic. The main reason was that there was a large portion of learners commenting positively while giving a negative rating for some unidentified reasons.

Results of both the negative and positive topic identification shed light on potential factors accounting for LMOOCs learners’ perceptions, satisfaction, and individual needs. Learners could be impressed by the friendly and innovative LMOOCs with quality teachers and learning content. Most learners were satisfied with the course, teachers, learning content, and materials. In addition, instructors’ good pronunciation, clear explanation, detailed instruction, and interaction with learners could motivate learner engagement and efficacy.

### Learners’ negative concerns across different course types

**Fig 6:**
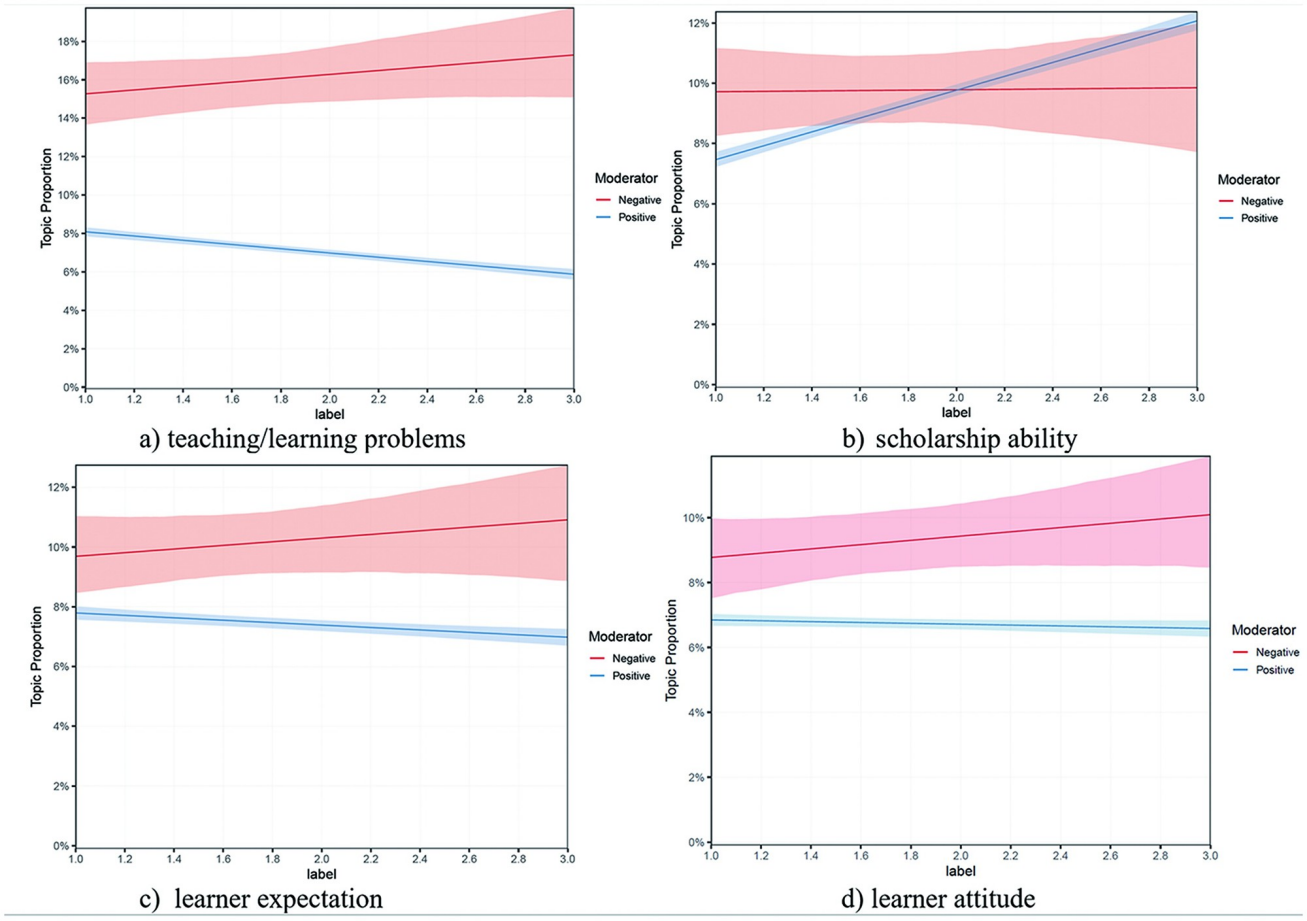
Moderating effects of course types.

The study examined the variations in four negative topics’ proportions across course types (Fig 6), with the x- and y-axes indicating course types and expected negative topic proportions. The blue and red lines represent positive and negative reviews, with the shades depicting the 95% confidence interval.

Results evidently demonstrated that the prevalence of three out of four negative topics (teaching problems, learner expectation, and learner attitude) in negative reviews increased as the course type increased. For instance, teaching problems’ negative prevalence rose from around 15% (Level = 1) to 17% (Level = 3). On the contrary, its positive prevalence had a significant decrease alongside the increased level. The variation of the topic of learner expectation shared a similar situation with teaching/learning problems. The variable of course type showed little influence on a positive learner attitude but a stronger influence on a negative attitude. On the contrary, the prevalence of positive reviews on the “scholarship ability” topic increased significantly alongside the increased course level while the negative one varies a little. The above results indicated that the major sources of LMOOCs learners’ negative perceptions and concerns were teaching/learning problems and learner expectations. For low-level courses, learners complained more about scholarship ability while for advanced courses “teaching/learning problems” and “learner expectation” were the main causes for negative evaluation.

## Discussion

Based on quantitative words’ frequency and co-occurrence analysis, comparative keywords analysis, and structural topic modeling, this study mined and visualized 69,232 course reviews from Chinese LMOOCs learners. The results revealed that students’ overall perception and attitude were positive, which was consistent with previous findings [19,23]. Most of the comments suggested that courses are good, suitable, and supportive for their language learning. The overwhelming proportion of positive comments may be explained by the present fact that LMOOCs were mainly offered to supplement formal education in colleges [28]. In general, LMOOCs serve as a significant instructional resource in the age of big data and information technology, offering learners a favorable learning experience, which aligns with the results from the studies [20,21]. Although the good experience and popularity of LMOOCS do not necessarily equate to a successful high-quality course [5], it provides a prerequisite for possibly effective language learning and lays a foundation for learners’ participation, engagement, and low dropout rates.

The STM analysis clustered 12 topics, among which 8 topics were found to be positive and 4 were negative, which were highly relevant to the evaluation of quality in LMOOCs. Learners’ positive concerns were mainly about the instructor’s teaching methods, high-quality content, learner improvement, and learner experience, which are possible key positive factors affecting learners’ effective language learning. In Peng and Jiang’s [23] sentiment analysis, they identified several positive categories including course content, overall LMOOC quality, lecture videos, and assignments and tests. Course content and quality could be considered as one main strength of LMOOCs as Martín-Monje and Borthwick proposed [29]. Our findings also revealed different important topics of teaching methods, learner improvement, and experience. For teaching methods, teachers need to shift from a traditional classroom mindset and embrace an online social one [11] by implementing innovative teaching techniques. And a salient factor related to speech style, native-like pronunciation, and appropriate speaking pace can significantly impact learners’ motivation and engagement, which was consistent with Peng and Jiang’s finding. For learner improvement, LMOOCs provided supplementary videos, learning materials, and solution explanation to enhance language skills, professional knowledge, and academic ability for on-campus classroom learners. And for learner experience, learners emphasized that they had an enjoyable, more student-centered learning process. Although some authors have noted that using MOOC platforms may make language learning a less enjoyable and/or productive experience [29], this study arrived at an opposite conclusion and regarded LMOOCs as more of an opportunity than a hindrance.

Learners’ negative concerns lie in three aspects (teaching/learning problems, learner expectation, and academic proficiency), which associate significantly with how to improve and optimize the quality of LMOOCs. For instance, for technical problems, teachers or LMOOCs platform managers may use artificial intelligence technology to add video subtitles automatically and improve the video quality, which will help reduce the difficulty and ensure the quality of teaching. For teaching management problems, learners are concerned with the aspects involved in the implementation of assignments and teachers’ feedback, peer evaluation, and time arrangement and difficulty level of the final exam. Besides, LMOOCs teachers provided language learners with relatively low “support for teaching, especially interactive support”, which is in line with the previous study’s findings [17]. Meanwhile, learners “expect” more personalized support services, a more stable learning platform including the Internet and material storage, and more idealized gains. LMOOCs should not follow a linear and rigid learning trajectory and should offer more self-paced courses to give learners more liberty to skip and move forward as they expect [30]. In general, our results are mainly consistent with Luo and Ye’s quality criteria framework of LMOOCs from learners’ perspectives, which consists of criteria of teacher criteria, teaching content, pedagogical criteria, technological criteria, and teaching management criteria [22].

Among the negative topics, we found “scholarship ability” (or academic proficiency) was a new and impressive topic that was not identified in previous studies. The negative evaluation of academic proficiency by foreign language MOOC learners indicates that the courses do not adequately fulfill their needs in this aspect. For instance, in academic English courses, language learners tend to make comments in English, and the length of their comments was significantly larger than that in Chinese. On one hand, it indicates a trend of learners’ strong alignment with the course content and requirements. On the other hand, learners’ demand for scholarship ability in special-purpose courses is different from that of ordinary language skill courses. This may be due to the fact that in the context of high employment pressure, solely evaluating language proficiency may not result in ideal job prospects. Instead, students tend to focus on pursuing postgraduate studies or government positions, where academic ability can assist them in achieving these goals. Therefore, foreign language teachers should be inspired by this finding and pay extra attention to the design of relevant courses to meet learners’ needs. As one learner wrote, “the learning process is both ‘painful’ and ‘meaningful’; ‘painful’ refers to the need to constantly take in new knowledge during the course and challenge themselves to complete various tasks; ‘meaningful’ means the course is not a bird course which can really help me a lot in my academic writing skills, information retrieval skills, logical thinking skills, and my further study!”

In addition, the negative topics’ prevalence is moderated by the course types from basic skill-oriented courses to advanced courses for special purposes. The moderator of course type should not be ignored in the implementation of LMOOCs. It could be a potential factor to explain the compatibility of LMOCCs and skill or practice-based language learning. Instructors should consider course type as an important factor for their course design, teaching pedagogy, and classroom management. It is not beneficial to discuss suitability diachronically without considering different course types [31]. Chen et al. [32] found that learner complaints are moderated by MOOCs grades and learners’ main complaints about high-graded MOOCs include problem-solving and practices whereas learners of low-graded courses are frequently annoyed by grading and course quality problems. For LMOOCs learners, we got an opposite finding with Chen et al. [32] which implies that in advanced courses learners are tending to be more critical about trivial problems and in language skill courses learners are expecting to learn more professional knowledge. This finding could be counterintuitive and should be cautioned by both language teachers and researchers and need to be further testified and explored.

## Practical implication

This study is the first attempt to combine frequency analysis, keyword analysis, and structural topic modeling to investigate learners’ perceptions, concerns, and experience in LMOOCs, which are largely under-investigated in the emerging field. The findings of this study could provide valuable insights for LMOOCs instructors to refine their teaching and for developers to design quality platforms and logistics involved to cater to students’ needs. These practical implications are significant for the development and implementation of LMOOCs.

Given the widespread large-scale comments on LMOOCs course platforms, instructors, content, and learning experiences, there is an opportunity to leverage these course reviews effectively. The approach used in this study could be applied to design and implement an automated system that analyzes and visualizes course reviews. Such a system could track learners’ learning status, satisfaction, and experiences, supporting teachers in adjusting teaching content, progress, and pedagogy, as well as aiding platform managers in making decisions to optimize the platform. Additionally, the system could easily integrate sentiment analysis functionality for more impactful results.

The study has identified several negative topics that are important to effective language learning, such as teaching/learning problems, academic writing, and learner expectation, which should be incorporated into the process of course implementation, and be addressed by instructors to ensure learners’ satisfaction and retention. For instance, instructors should improve the course materials, identify knowledge gaps, and tailor their instruction to better meet learners’ needs. Additionally, instructors have to identify areas where learners need more support, such as with difficult concepts or technical issues. Targeted resources and support are necessary to help learners overcome these challenges to increase learners’ motivation and engagement in the course. They can identify the methods that are most effective and make changes to their teaching to improve the learning experience. Given students’ concerns about the trivial related to learning problems, such as the quantity of assignments, assessment, videos problem, teachers’ pronunciation, and reading presentation slides, instructors may allow sufficient time for completing assignments and assessments. Options of having no subtitles, second language subtitles, or bilingual subtitles are best made available to learners. Instructors also need to avoid speech accents and adjust their teaching styles to meet learners’ expectation.

This study highlights the role of course type in moderating the prevalence of negative topics. Instructors need to consider the course type and tailor the course content, teaching pedagogy, and classroom management accordingly. For example, the study emphasizes the importance of promoting learners’ scholarship ability in addition to their language skills. This can increase their motivation to continue language learning and enhance their job prospects. LMOOCs instructors and researchers need to shift their focus from the suitability of LMOOCs to external factors such as course type and learner grade, which can influence the course content’s adjustment. It is possible that there may not be any suitability challenge between MOOCs technology and language learning, but determining which type of course is suitable or not is critical.

## Conclusion

In conclusion, our study sheds light on how learners perceive LMOOCs and their major concerns, providing actionable suggestions for LMOOCs implementation. To enhance learner attitude, satisfaction and experience, LMOOCs platforms can leverage text mining and visualization methods to capture the dynamic nature of learner demands, experiences, and course reviews feedback. Moreover, integrating external factors such as learners’ individual characteristics, course type labels, and even learner grades into text mining models can help trace learners’’ evolving perceptions and provide teachers with a comprehensive understanding of LMOOCs’ strengths, weaknesses, and even nature. Despite its rich findings, this study has two aspects of limitations. The first issue concerns data representativeness, which were collected from the Chinese LMOOCs learners, and thus the results should not be extrapolated as representing all LMOOCs learners. The other is that the division criteria for positive and negative reviews are heavily dependent on learners’ ratings where some learners’ comments are positive but the factual rating is negative. These limitations could lead to a biased and inaccurate conclusion. Future research could be conducted from the following perspectives. First, we will use more learners’ reviews from other influential LMOOCs platforms like *UMOOCs* in China, *edX*, and *coursera* to replicate this study. Second, it’s more reasonable to improve Quanteda’s word-based algorithm by considering 2- or 3-gram phrases as basic features and examining the distribution of the key phrases. Last, it is suggested to consider more covariates like learner characteristics to produce more fine-tuned insights.
